# Envelopes found early after acquisition compared to those in the chronically infected partner do not have enhanced alpha4 beta7 binding or utilization

**DOI:** 10.1186/1742-4690-9-S2-P149

**Published:** 2012-09-13

**Authors:** B Etemad, A Redd, D Serwadda, T Lutalo, S Reynolds, R Gray, T Quinn, M Sagar

**Affiliations:** 1 Brigham and Women’s Hospital, Harvard Medical School , Cambridge, MA, USA; 2 Lab of Immunoregulation, NIAID/ NIH, Baltimore, MD, USA; 3 Makerere University, Kampala, Uganda; 4 Johns Hopkins University School of Medicine, Baltimore, MD, USA; 5 Johns Hopkins University Bloomberg School of Public Health, Baltimore, MD, USA; 6 Brigham and Women’s Hospital affiliated of Harvard Medical School , Cambridge, MA, USA

## Background

It has been hypothesized that the RV144 immune correlate, V1V2 antibodies, block binding to gut homing receptor, alpha4 beta7, which may limit virus access to gut associated lymphoid tissue. We hypothesized that if alpha4 beta7 binding is important during HIV-1 acquisition, viruses with envelopes isolated from newly infected subjects should have enhanced ability to bind the alpha4 beta7 integrin and/or infect cells with high levels of alpha4 beta7 receptor compared to variants present in the chronically infected transmitting partner.

## Methods

Envelopes isolated from newly infected subjects and the transmitting partner were incorporated into NL4-3. Virus stocks were generated from peripheral blood mononuclear cell cultures. CD8+ and CD4+ T cells were activated with PHA, IL-2, and retinoic acid (RA). Binding was examined with quantitative PCR, and replication was assessed by estimating produced infectious virus on TZM-bl cells. Comparisons were done using Wilcoxon matched-pairs signed rank test.

## Results

Samples from 9 newly infected subjects were collected a median of 93 days after estimated HIV-1 seroconversion (range 17 – 324 days), and all the transmitting partners had chronic persistent infection of more than two years duration at the time of estimated transmission. The alpha4 beta7 receptor expression increased in both CD8+ and CD4+ T cells by day 6. Viruses with recipient (median 9 copies, range 0 – 632) versus donor (median 9 copies, range 0 – 313) demonstrated no significant difference (p = 0.9) in binding to alpha4 beta7 expressing CD8+ T cells. Recipient viruses also demonstrated similar replication in CD4+ T cells compared to the donor envelope viruses (p = 0.4).

## Conclusion

Enhanced alpha4 beta7 binding does not distinguish envelopes found early after acquisition compared to those circulating in chronically infected partners. Thus, the V1V2 antibodies found as a RV144 immune correlate likely does not provide protection by blocking access to the alpha4 beta7 integrin.

